# The genetic basis of music ability

**DOI:** 10.3389/fpsyg.2014.00658

**Published:** 2014-06-27

**Authors:** Yi Ting Tan, Gary E. McPherson, Isabelle Peretz, Samuel F. Berkovic, Sarah J. Wilson

**Affiliations:** ^1^Melbourne Conservatorium of Music, University of MelbourneParkville, VIC, Australia; ^2^International Laboratory for Brain, Music and Sound Research and Department of Psychology, Université de MontréalMontreal, QC, Canada; ^3^Department of Medicine, Epilepsy Research Centre, University of MelbourneHeidelberg, VIC, Australia; ^4^Melbourne School of Psychological Sciences, University of MelbourneParkville, VIC, Australia

**Keywords:** music, music ability, music perception, music production, genetic, genome, review

## Abstract

Music is an integral part of the cultural heritage of all known human societies, with the capacity for music perception and production present in most people. Researchers generally agree that both genetic and environmental factors contribute to the broader realization of music ability, with the degree of music aptitude varying, not only from individual to individual, but across various components of music ability within the same individual. While environmental factors influencing music development and expertise have been well investigated in the psychological and music literature, the interrogation of possible genetic influences has not progressed at the same rate. Recent advances in genetic research offer fertile ground for exploring the genetic basis of music ability. This paper begins with a brief overview of behavioral and molecular genetic approaches commonly used in human genetic analyses, and then critically reviews the key findings of genetic investigations of the components of music ability. Some promising and converging findings have emerged, with several loci on chromosome 4 implicated in singing and music perception, and certain loci on chromosome 8q implicated in absolute pitch and music perception. The gene *AVPR1A* on chromosome 12q has also been implicated in music perception, music memory, and music listening, whereas *SLC6A4* on chromosome 17q has been associated with music memory and choir participation. Replication of these results in alternate populations and with larger samples is warranted to confirm the findings. Through increased research efforts, a clearer picture of the genetic mechanisms underpinning music ability will hopefully emerge.

## Introduction

Music is ubiquitous in all known human cultures. The general capacity for human beings to perceive, produce, and enjoy music even in the absence of formal music training suggest that music may be “hardwired” in our genetic makeup. However, the diversity of music competency across individuals adds impetus to the long-standing debate of whether musicians are born or made.

Studies on the genetic basis of music ability have been relatively scarce, compared with more extensive investigation conducted in the language domain (for recent reviews of the genetic findings on speech and language, see Newbury and Monaco, 2010; Carrion-Castillo et al., 2013; Graham and Fisher, 2013; Raskind et al., 2013; Szalontai and Csiszar, 2013). Moreover, the earlier behavioral genetic investigations of music ability often lacked scientific rigor or suffered from small sample sizes (see Coon and Carey, 1989 for examples). The advent of molecular genetics in the post-genomic era holds much promise for this relatively underexplored field.

Since the boom of molecular genetic research, the current state of knowledge of the genetic basis of music ability has not been reviewed. Thus, it is timely to consolidate behavioral and molecular genetic findings and provide a critical overview of what is currently known about the genetic basis of various music phenotypes. Current challenges and possible directions for future research are also considered. This review aims to facilitate a greater understanding of this relatively new field among music and genetics researchers, and encourage increased research effort into uncovering the genetic basis of music ability.

## Human genetic methods: overview

The relationship between phenotypes and genes can be investigated through various genetic analytical approaches. In order to offer readers of diverse backgrounds a preliminary understanding, this section provides an introductory overview of the behavioral and molecular genetic approaches commonly used in human genetic analyses. A glossary of genetic terminology (terms shown in bold in the text) is also available as Supplementary Material online.

### Behavioral genetic approaches

#### Familial aggregation

One of the first questions asked in human genetic analysis is whether a trait clusters in families above chance level. **Familial aggregation** can address this question by comparing whether the prevalence of a trait is higher within the family of a **proband** than that in the general population (Naj et al., 2012). This approach is non-invasive because only phenotypic information is gathered from the families and controls. One common measure arising from familial aggregation is the **sibling recurrence-risk ratio (λ_s_)**, which determines the proportion of proband siblings also exhibiting the studied trait, relative to the population prevalence. The magnitude and patterns of familial correlations observed in familial aggregation studies can yield useful clues to the roles played by genes and the environment (Naj et al., 2012). While a λ_s_ close to 1 suggests genetic influences are unimportant, higher values (>5) indicate that a genetic hypothesis is worth pursuing (Mitry et al., 2011). A related measure is the **sibling relative risk (sib RR)**, which is the ratio of the proband λ_s_ to the control λ_s_.

Familial aggregation measures such as λ_s_ may be inflated by **ascertainment bias** (Guo, 1998). Moreover, familial aggregation only serves to determine the existence of familial clustering. It does not seek to explain how much of the familial clustering is due to genetic or environmental factors. Such questions can be addressed through follow-up studies, such as twin and adoption studies.

#### Twin studies

Twin studies take a step further by disentangling the relative contributions of genetic and environmental factors on trait variation (Verweij et al., 2012). **Monozygotic** (MZ) twins share 100% of their genes whereas **dizygotic** (DZ) twins share on average 50% of their genes. By comparing the similarity of the MZ twin pairs on the trait of interest with that of the DZ twin pairs, greater similarity exhibited by MZ twin pairs indicates a possible genetic influence. In other words, if the **concordance** for a trait is much higher in the MZ twins, the trait is likely to have significant **heritability**.

Through twin studies, the effects of genetic influence, shared environment (family environment), and unique environment on a trait can be estimated through structural equation modeling using statistical programs such as *Mx* (Neale et al., 2006). Basic twin designs can be extended to allow multiple traits to be studied simultaneously or to analyze more complex genetic and environmental influences. For instance, additional family members can be incorporated into the design (Verweij et al., 2012).

There are some criticisms surrounding the validity of twin studies given that the twin design is built on a number of assumptions (Richardson and Norgate, 2005). For instance, the “equal environment” assumption presupposes that regardless of zygosity, all twins raised together experience equally similar shared environments. Some research, however, suggests that MZ twins may be treated more similarly than DZ twins (Plomin et al., 1976; but see Borkenau et al., 2002).

Besides twin studies, heritability can also be estimated from family pedigrees. **Heritability in the narrow sense (*h*^2^)** is the ratio of the **additive genetic variance** to the total phenotypic variance of a trait. Since the magnitude of *h*^2^ can indicate the statistical power for discovering the causal genes of a trait, heritability estimation serves as a good precursor to molecular genetic studies (Bochud, 2012). If the *h*^2^ estimates of multiple related traits are available, the trait with the best *h*^2^ estimate can be chosen for subsequent gene mapping. In human research, the general consensus is that *h*^2^ estimates below 0.2 are considered low, those between 0.2 and 0.5 are moderate, and estimates above 0.5 indicate high heritability. High *h*^2^ estimates suggest that the **genotype** is closely correlated with the trait **phenotype**, but it should be noted that this does not necessarily imply that every gene associated with the trait has a large effect on the phenotype (Visscher et al., 2008). Some limitations of heritability estimation include a lack of information on the **mode of inheritance** of the trait, and the possibility that *h*^2^ estimates may vary across populations or with time. In large pedigrees, there may also be cohort effects across different generations living under different socioeconomic circumstances, which may confound the “equal environment” within the sample population (Bochud, 2012).

#### Segregation analysis

After estimating the heritability of a trait of interest, the mode of inheritance of the trait can be elucidated through **segregation analysis**. Different segregation models representing various inheritance patterns of the trait are fitted to the family data. Using maximum likelihood procedures, the genetic model of best fit (i.e., the inheritance pattern which best explains how the trait is transmitted down the family line) can be identified. An important measure from segregation analysis is the **segregation ratio**, which is the proportion of offspring who inherit the trait of interest from a parent (Strachan and Read, 1999). The expected segregation ratios for **autosomal dominant inheritance** and **autosomal recessive inheritance** are 0.5 and 0.25, respectively. As these expected segregation ratios are for **Mendelian traits**, deviation from these values indicate that the trait of interest may have incomplete **penetrance**, be predisposed by several genes in different loci, or that it is determined by both genetic and environmental factors. For instance in a study by Theusch and Gitschier (2011), absolute pitch was reported to have a segregation ratio of 0.089, which suggests that the trait is not inherited in a simple Mendelian fashion. Similar to familial aggregation, a potential problem with segregation analysis is ascertainment bias, which may inflate the segregation ratio (Strachan and Read, 1999; Nandram et al., 2011), however it is possible to statistically correct for this (Li and Mantel, 1968; Li et al., 1987; Yao and Tai, 2000; Nandram et al., 2011).

Segregation analysis often serves as a precursor to **parametric linkage analysis**, as the latter requires the inheritance pattern of the studied trait to be specified (Schnell and Sun, 2012).

### Molecular genetic approaches

#### Linkage analysis

Once the genetic basis of a trait has been established using some of the above-mentioned methods, the next step is to conduct linkage analysis to map the potential genetic **loci** predisposing the trait. Linkage analysis requires each family member of a large family, or of several family pedigrees to be genotyped, typically using **single-nucleotide polymorphism (SNP) arrays**. If a genetic **locus** is thought to predispose a trait, family members who share the same **markers** near this locus should exhibit greater trait resemblance compared to those who do not share the markers. Therefore, linkage analysis aims to identify the markers on the SNP array that are commonly present in participants within or across pedigrees who exhibit the trait of interest. It should be noted that the identified markers are not the actual genes predisposing a trait; they are merely known locations on the **genome** that are near the gene of interest (Carey, 2003a). **“Linkage”** is said to occur when two gene loci that are in close proximity on the same **chromosome** are inherited together. In other words, if a marker is commonly shared among family members exhibiting the trait of interest, this marker is likely to be in linkage with the actual locus of the trait.

There are two common approaches to estimate linkage. The **non-parametric or model-free linkage analysis** basically tests whether relatives exhibiting the trait of interest share more **alleles** than would be expected by chance (Xu et al., 2012). In contrast, **parametric or model-based linkage analysis** requires the specification of the mode of inheritance, as ascertained by segregation analysis. Linkage is then tested by comparing the probability of obtaining the current test data if a marker locus and the trait locus are linked, to the probability of obtaining the test data if the two loci are not linked (Schnell and Sun, 2012). The ratio of the two probabilities expressed on a logarithmic scale is known as the **LOD score**. LOD scores are computed across all markers for each pedigree and then summed across different pedigrees. A LOD score ≥3 is typically considered significant evidence for linkage, as it indicates that the odds that the two loci are linked and inherited together are greater than 1000–1. Alternatively, a LOD score ≤ −2 is considered significant evidence to reject linkage. In general, scores within the range of −2 < *x* < 3 are regarded as inconclusive evidence for linkage, with those between 2 ≤ *x* <3 warranting additional study.

Linkage analysis is very successful in identifying Mendelian traits with a simple mode of inheritance, such as Huntington's disease. It has been less effective for complex traits predisposed by multiple genes as each gene individually exerts only a small effect on the trait (Nsengimana and Bishop, 2012). Large sample sizes are therefore needed to obtain adequate statistical power to detect linkage. Statistical power can also be increased by performing multipoint analysis (i.e., using multiple markers simultaneously), allowing more precise identification of the trait locus (Lathrop et al., 1984).

As parametric linkage analysis requires the pattern of inheritance to be specified, misspecification of the genetic model may lead to loss of power (Schnell and Sun, 2012). While this potential problem can be alleviated by testing more than one genetic model, multiple-testing issues increase the likelihood of false positives (Weeks et al., 1990). A further shortcoming of linkage analysis is that the identified linkage region typically contains numerous genes. Fine-mapping of the linkage region is therefore necessary to narrow down the locus and determine the possible causative gene (Carey, 2003b). With the development of exome sequencing technologies (see below), fine-mapping is now less essential as all genes in the linkage region can be quickly analyzed.

#### Association analysis

Association analysis is a statistical method used to investigate the association between a **genetic variant** and a trait (Carey, 2003b). Association analysis can be used to test potential genetic variants that lie in significant linkage regions. It can also be employed when there are well-founded reasons to suspect a gene's involvement in predisposing a trait. An association analysis can either adopt a **population-based design** involving unrelated cases and controls, or a **family-based design** in which relatives of cases serve as controls for the study.

In the candidate gene approach, statistical tests are performed to determine if the cases have a higher frequency of a particular **allelic variant** of the **candidate gene**, as compared with the controls. The possible association between an allelic variant and the trait of interest may yield valuable information about the variant's role in the biological pathway of the trait. One major limitation of candidate gene studies is that only specific allelic variants are investigated; the success of an association study thus hinges on the accuracy of a researcher's “educated guess” of the potential candidate genes. Moreover, if the trait of interest is complex and multiple genes are involved, the candidate gene approach is not able to detect the influence of the predisposing genes in other loci.

By contrast, a genome-wide association study (GWAS) can be conducted without prior knowledge of potential candidate genes. It involves an “agnostic” or non-candidate driven search of the entire human genome, typically using SNP arrays with a large number of common SNP markers found throughout the genome (Sun and Dimitromanolakis, 2012). If certain SNPs have a higher incidence in the cases relative to the controls, this will indicate a possible association of these SNPs with the trait of interest.

GWAS is a useful approach to find novel candidate genes predisposing a trait of interest, especially if the biological pathway of the trait is not well-understood. It is also possible to identify multiple genetic contributors to a complex trait, even if each of these may be conferring only a small effect (Reich and Lander, 2001). The downside is that to identify such SNPs of low effect size, large sample sizes are needed to achieve adequate power (typically thousands or even tens of thousands of cases and controls) (Spencer et al., 2009). However, large sample sizes may give rise to confounding factors such as **population stratification** and **cryptic relatedness** that result in false positives (Nsengimana and Bishop, 2012). Moreover, GWAS does not identify complete genes; it only identifies the genomic regions possibly associated with the trait, which in some cases may not even have a protein-coding gene in the vicinity.

A common problem faced by both the candidate gene approach and GWAS is the minimal replication of significant results across association studies (Lewis and Knight, 2012). Researchers must therefore exercise caution and critically examine the validity of published association studies, and make prudent design decisions for their own association studies (Attia et al., 2009).

#### Exome sequencing

With the advancement of high-throughput **next-generation sequencing (NGS)** technologies, exome sequencing has emerged as a rapid and cost-effective method for human genetic analysis (Singleton, 2011). The **exome** is the portion of the genome containing protein-coding information and represents about 1.5% of the whole genome. Exome sequencing is based on the assumption that changes in **exons** may modify the function of proteins coded by exons, thereby leading to changes in the phenotype. By targeting the exome instead of the entire genome, exome sequencing presents a cost- and time-effective means to find possible genetic variants predisposing a trait. Exome sequencing uses **sequence capture methods** to selectively capture exons from a **DNA** sample and enrich the available information from each sample, before using high-throughput sequencing to identify coding variants in the exome (Ku et al., 2012).

Since the publication of the first proof-of-concept study which used exome sequencing to identify causal variants for a rare Mendelian disorder (Ng et al., 2009), exome sequencing has shown much promise in discovering coding variants for both Mendelian and non-Mendelian traits (Singleton, 2011). Like GWAS, exome sequencing can be performed without the foreknowledge of potential candidate genes or genetic variants. Its usefulness has also been shown in the diagnosis of disorders characterized by **genetic heterogeneity** (Singleton, 2011). Another advantage of exome sequencing is that it can identify uncommon causal variants predisposing rare Mendelian traits, which cannot be identified through linkage studies due to inadequate study power (Singleton, 2011).

Ultimately, exome sequencing may become a valuable genetic screening and diagnostic tool, allowing robust diagnoses to be reached rapidly and cost-effectively (Ku et al., 2012). However, it has some limitations. For instance, some regions of interest in the exome are difficult to sequence with the current technology, and the large amount of sequencing data generated poses computational challenge for analysis. Moreover, exome sequencing is unable to detect large deletions or rearrangements in the genome, and there is a major risk of false positives due to difficulties determining the biological relevance of coding variants to the trait of interest.

#### Copy number variation (CNV) analysis

**Copy number variants** are structural variants (>1000 **base pairs**) whose **copy numbers** deviate from those found in the human reference genome. Such variation in the genome (known as copy number variation) includes insertions, deletions, duplications, inversions and translocations (Wain et al., 2009). More than 300 known causal genes for diseases are found to overlap with CNVs (Sebat et al., 2004), highlighting the impact CNVs may exert on genetic mechanisms and phenotypic variation. Understanding the functional impact of CNVs, therefore, offers a fruitful means of elucidating the genetic basis of complex phenotypes.

There are several approaches to CNV detection. **Microarray**-based CNV analysis techniques typically use SNP arrays or **aCGH (array comparative genomic hybridization)** platforms to detect copy number gains or losses in the test sample compared with a reference sample (Alkan et al., 2011). CNV detection can also be achieved through a sequencing-based approach.

CNV analysis has shown its utility in the diagnosis of syndromes with **heterogeneous phenotypes** (Coughlin et al., 2012). However, understanding the significance and effect of detected CNVs on a trait of interest remains challenging. In addition, microarray-based CNV analysis has a number of drawbacks. Copy neutral alterations (such as inversions and translocations) that do not cause a change in the total amount of genetic material cannot be identified by the microarray-based approach, even though such alterations are potentially deleterious. The microarray-based approach is also less sensitive in detecting duplications than deletions.

Given the continued advancement in sequencing technologies, sequencing-based approaches are likely to supersede microarray-based CNV analysis. In time, the ability of NGS to detect various forms of genetic variants (including small insertions/deletions and copy neutral variants) will improve and the cost of massive parallel sequencing will become less expensive. Nevertheless, this approach has not yet reached maturity and standard protocols and measures for sequencing-based CNV analysis have not been established. Moreover, the bioinformatics infrastructure, support, and cost required for managing, analyzing, and storing large amounts of sequencing data need to be carefully considered (Teo et al., 2012). In addition, the relatively short read lengths of NGS pose mapping issues in aligning the sequenced reads with the reference genome, especially in the duplicated regions of the genome. Thus, as with a microarray-based approach, copy number duplications remain less detectable than deletions with the sequencing-based method. Longer read lengths afforded by improvements in sequencing technology will potentially mitigate this issue.

## Scope of the review

Indexed searches were performed in Scopus by searching for the following keywords: music^*^ and (gene or genetic or genome or heritability or hereditary or innate) in the keyword field. Terms such as music therapy, algorithm, dystonia, musicola, animal, bird, and songbird were excluded. Subject areas such as computer science, engineering, health professions, physics and astronomy, mathematics, nursing and business, management and accounting were also excluded. Only papers published in English between 1988 and 2014 were considered since earlier genetic studies were often fraught with methodological limitations, such as small sample sizes and lack of scientific rigor. Based on these criteria the search identified 97 articles that were then manually canvassed to select those directly addressing the genetic bases of various aspects of music ability using behavioral genetic or molecular genetic methods. In addition, the reference section of each article was examined to identify additional studies not captured in the initial search. In total, this process identified 21 papers included in this review.

## Summary of the review findings

Table 1 shows the various music traits investigated and the number of genetic studies conducted on each trait to date. It indicates that the majority of studies have focused on music perception abilities (74%), of which absolute pitch has been most extensively investigated (39%). In light of this, findings pertaining to music perception abilities are examined first, followed by those relating to music production abilities. Tables 2, 3 that follow provide a more detailed summary of the genetic findings from behavioral and molecular studies, respectively.

**Table 1:** **The number of genetic studies investigating various music traits**.

**Music trait**		**Studies**	**Authors**
Music perception	Absolute pitch	9	Profita and Bidder, 1988; Gregersen and Kumar, 1996; Baharloo et al., 1998, 2000; Gregersen et al., 1999, 2001, 2013; Theusch et al., 2009; Theusch and Gitschier, 2011
	Congenital amusia	1	Peretz et al., 2007
	Music perception (in general)	5	Drayna et al., 2001; Pulli et al., 2008; Ukkola et al., 2009; Ukkola-Vuoti et al., 2013; Oikkonen et al., 2014
	Melodic memory	1	Granot et al., 2007
	Music listening	1	Ukkola-Vuoti et al., 2011
Music production	Singing accuracy	1	Park et al., 2012
	Singing participation	2	Coon and Carey, 1989; Morley et al., 2012
	Music creativity	2	Ukkola et al., 2009; Ukkola-Vuoti et al., 2013
Self-reported music ability		1	Vinkhuyzen et al., 2009

**Table 2:** **Summary of behavioral genetic studies investigating various music traits**.

**Trait**	**Type of study**	**Participants**	**Findings**
Absolute pitch (AP)	Family segregation analysis	35 AP probands from 19 families	SR: 0.24–0.37
	(Profita and Bidder, 1988)		
	Familial aggregation	101 AP probands	λ_s_: ~20
	(Gregersen and Kumar, 1996)		
	Familial aggregation	92 self-reported AP probands	λ_s_: ~7.5
	(Baharloo et al., 1998)	520 non-AP probands	(controlled for early music training)
	Familial aggregation	2707 tertiary music students	Sib RR: ~8.3
	(Gregersen et al., 1999)		
	Familial aggregation	74 AP-1 probands with 113 siblings	λ_s_: 7.8–15.1
	(Baharloo et al., 2000)	625 controls	(controlled for early music training)
	Familial aggregation	1067 music theory students	Sib RR: ~12.2
	(Gregersen et al., 2001)		
	Family segregation analysis	1463 families with AP-1 probands	SR: 0.089
	(Theusch and Gitschier, 2011)		
	Twin study	14 MZ and 31 DZ female twin pairs	CCR_MZ_: 78.6%; CCR_DZ_: 45.2%
	(Theusch and Gitschier, 2011)		
Congenital amusia	Familial aggregation	13 probands, 58 family members (9 families)	λ_s_: 10.8
	(Peretz et al., 2007)	17 controls, 58 family members (10 families)	λ_o_: 2.3
Melodic perception	Twin study	136 MZ and 148 DZ twin pairs	*A*: 71–80%; *C:* 0%; *E:* 20–29%
(tune recognition)	(Drayna et al., 2001)		
Perception of pitch and rhythm	Pedigree study	15 Finnish musical families (*N* = 234)	Heritability estimates for KMT: 42%; SP: 57%; ST: 21%; COMB: 48%
	(Pulli et al., 2008)		
	Pedigree study	19 Finnish musical families (*N* = 343)	Heritability estimates for KMT: 39%; SP: 52%; ST: 10%; COMB: 44%
	(Ukkola et al., 2009)		
	Pedigree study	76 Finnish families (*N* = 767)	Heritability estimates for KMT: 46%; SP: 68%; ST: 21%; COMB: 60%
	(Oikkonen et al., 2014)		
Singing	Pedigree study	73 extended families (*N* = 1008)	Heritability: 40%
(pitch accuracy)	(Park et al., 2012)		
Participation in singing activities	Twin study	850 twin pairs	*A*: 71% (males) & 20% (females);
	(Coon and Carey, 1989)	(from Loehlin and Nichols, 1976)	*C*: 8% (males) & 59% (females)
Music creativity	Pedigree study	19 Finnish musical families (*N* = 343)	Heritability for music creativity: 84%
	(Ukkola et al., 2009)		(Composing: 40%; Arranging: 46%; Improvising: 62%)
Self-reported music ability	Twin study	850 twin pairs	Out-of-school music performances:
	(Coon and Carey, 1989)	(from Loehlin and Nichols, 1976)
			*A*: 38% (males) & 10% (females);
			*C*: 18% (males) & 63% (females)
	Twin study	1685 Netherlands twin pairs	Musical aptitude:
	(Vinkhuyzen et al., 2009)	(12–24 years)	*A*: 66%; *C:* 8%; *E:* 25% (males)
			*A*: 30%; *C:* 54%; *E:* 16% (females)
			Exceptional musical talent:
			*A*: 92%; *C:* 0%; *E:* 8%

*λ_s_, sibling recurrence risk; λ_o_, offspring recurrence risk; CCR_MZ_, casewise concordance rate for MZ twins; CCR_DZ_, casewise concordance rate for DZ twins; COMB, Combined music aptitude (SP+ST+KMT) score; A, additive genetic variance (heritability); C, common/shared environmental variance; E, unique environmental variance; KMT, Karma music test; SP, Seashore's pitch discrimination test; ST, Seashore's time discrimination test; Sib RR, sibling relative risk; SR, segregation ratio*.

**Table 3:** **Summary of molecular genetic studies investigating various music traits**.

**Trait**	**Study type**	**Participants**	**Ancestry**	**Locus implicated**	**Genetic variant(s) implicated**	**Gene implicated**	**Possible function(s) of the gene**
Absolute pitch (AP)	Genome-wide linkage study	73 AP families^*^	European,	8q24.21	SNP rs3057	*ADCY8*	Learning and memory
	(Theusch et al., 2009)		Ashkenazi Jewish,		LOD = 2.330 Eu/AJ/I		
			Indian,		LOD = 3.464 Eu		
			East Asian				
				7q22.3	SNP rs2028030		
					LOD = 2.074 Eu		
					LOD~1–1.5 E Asian		
				8q21.11	SNP rs1007750		
					LOD = 2.069 Eu/AJ/I		
					LOD = 2.236 for Eu		
				9p21.3	SNP rs2169325		
					LOD = 2.048 Eu		
	Genome-wide linkage study; exome sequencing	53 AP multiplex families,	Caucasian, Asian	6q14.1–16.1		*EPHA7*	Neural connectivity and development
	(Gregersen et al., 2013)	36 synaesthesia multiplex families		Peak LOD = 4.68			
				2q	SNP rs1482308		
							
					HLOD = 4.7		
					(combined data set)		
					SNP rs6759330		
					HLOD = 3.93		
					(AP families)		
Perception of pitch and rhythm	Genome-wide linkage study	15 musical families (*N* = 234)	Finnish	4q22	Near markers D4S423 and D4S2460	*UNC5C*	Netrin receptor.
	(Pulli et al., 2008)				LOD = 3.33		Netrins direct axon extension and cell migration during neural development
				8q13-21		*TRPA1*	Implicated in the hair cell transduction channel of the inner ear
				LOD = 2.29			
	Candidate gene association study	19 musical families (*N* = 343)	Finnish	12q14.2	RS1 + RS3 haplotype (corrected *p* < 0.001 for KMT and COMB)	*AVPR1A*	Social cognition and behavior; spatial memory
	(Ukkola et al., 2009)						
	Genome-wide CNV analysis	5 extended families,	Finnish	Deletion on 5q31.1 (linked to low COMB)		*Pcdha 1-9*	Neural migration, differentiation and synaptogenesis; learning and memory
	(Ukkola-Vuoti et al., 2013)	172 unrelated individuals					
				Duplication on 8q24.22 (linked to low COMB)		*ADCY8*	Learning and memory
	Genome-wide linkage and association study	76 families (*N* = 767)	Finnish	3q21.3	SNP rs9854612 (PPLD = 0.98 for COMB)	*GATA2*	Development of cochlear hair cells and the inferior colliculus
	(Oikkonen et al., 2014)						
				4p15-q24 (linked to SP, ST, KMT, COMB)	SNP rs13146789 & SNP rs13109270	*PCDH7*	Expressed in cochlea (chicken) & amygdala (mice)
					(PPLD = 0.81 for COMB)		
Music memory	Candidate gene association study	82 university students		12q14.2	RS1+RS3 haplotype	*AVPR1A*	Social cognition and behavior; spatial memory
	(Granot et al., 2007)						
				17q11.2	HTTLPR	*SLC6A4*	Reward-seeking behavior; neuropsychiatric conditions
Music listening	Candidate gene association study	31 families (*N* = 437)	Finnish	12q14.2	RS1+AVR haplotype: current active music listening (*p* = 0.0019);	*AVPR1A*	Social cognition and behavior; spatial memory
	(Ukkola-Vuoti et al., 2011)				RS1+RS3 haplotype: lifelong active music listening (*p* = 0.0022)		
Singing (pitch accuracy)	Genome-wide linkage and association study; exome sequencing; CNV analysis (Park et al., 2012)	73 families(*N* = 1008)	Mongolian	4q23	Marker D4S2986		
					LOD = 3.1		
				4q26	SNP rs12510781 (*p* = 8.4 × 10^−17^)	*UGT8*	Catalyzes the transfer of galactose to ceramide
					SNP rs4148254 (*p* = 8.0 × 10^−17^)		
				Deletion at 5.6 kb upstream of *UGT8* (linked to low pitch accuracy)			
Choir participation	Candidate gene association study	262 amateur choir singers,		17q11.2	STin2.9, STin2.12: choir singers (*p* = 0.04);	*SLC6A4*	Reward-seeking behavior; neuropsychiatric conditions
	(Morley et al., 2012)	261 controls			STin2.10: controls (*p* = 0.009)		
Music creativity	Genome-wide CNV analysis	5 extended families,	Finnish	Deletion on 5p15.33 (creative phenotype)		*ZDHHC11*	
	(Ukkola-Vuoti et al., 2013)	172 unrelated individuals					
				Duplication on 2p22.1 (creative phenotype)		*GALM*	Associated with serotonin transporter binding potential in human thalamus, which is implicated in music perception
				Deletion on 2p12, 3p14.1, 3q28 (non-creative phenotype)			

* *Families with at least two AP possessors who were not simply a parent-child relative pair*.

*ADCY8, adenylate cyclase 8; AJ, Ashkenazi Jewish ancestry; AVPR1A, arginine vasopressin receptor 1A; CNV, copy number variation; COMB, Combined music aptitude (SP+ST+KMT) score; E Asian, East Asian ancestry; EPHA7, ephrin type-A receptor 7; Eu, European ancestry; GALM, galactose mutarotase (aldose 1-epimerase); GATA2, GATA binding protein 2; HLOD, heterogeneity logarithm of odds score; I, Indian ancestry; KMT, Karma Music Test; LOD, logarithm of odds score; PCDH7, protocadherin 7; Pcdha 1-9, protocadherin alpha 1 to 9; PPLD, posterior probability of linkage disequilibrium; SLC6A4, solute carrier family 6 (neurotransmitter transporter, serotonin), member 4; SNP, single nucleotide polymorphism; SP, Seashore's pitch discrimination test; ST, Seashore's time discrimination test; TRPA1, transient receptor potential cation channel, subfamily A, member 1; UGT8, uridine diphosphate glycosyltransferase 8; UNC5C, UNC-5 homolog C (C. elegans); ZDHHC11, zinc finger, DHHC-type containing 11*.

### Music perception abilities

#### Absolute pitch ability

Absolute pitch (AP) or “perfect pitch” is the rare music ability of being able to identify or produce pitches without relying on an external reference. It has an estimated prevalence of less than 1 in 10,000 (Bachem, 1955; Profita and Bidder, 1988). While AP is neither a prerequisite nor a predictor for outstanding musicianship, its rarity and the musical advantages it confers have generated much research interest into its etiology.

Several studies have explored the genetic basis of AP through family studies. One of the earliest was a segregation study conducted by Profita and Bidder (1988), who reported significant familial incidence in 35 AP probands from 19 families. AP was more common in females of this sample, and **vertical transmission** was commonly observed. The segregation ratio was estimated to lie between 0.24 and 0.37, suggesting a possible autosomal dominant gene with incomplete penetrance. Recurrence risk ratios could not be determined because the study did not recruit any control participants.

A subsequent familial aggregation study yielded a sibling recurrence risk-ratio (λ_s_) estimate of 20, meaning that siblings of AP possessors are approximately 20 times more likely to possess AP relative to the general population (Gregersen and Kumar, 1996). Gregersen's team subsequently conducted another two familial aggregation studies and obtained sibling relative risk (sib RR) estimates of 8.3 and 12.2, respectively (Gregersen et al., 1999, 2001). This measure indicates that the siblings of the AP possessors were 8.3 and 12.2 times more likely to have AP compared to the siblings of the controls. Sib RR generally provides a more conservative measure than λ_s_ (Naj et al., 2012), likely accounting for the lower sib RR values. As noted in the previous section, familial aggregation does not distinguish between genetic and environmental contributions to a trait, making it possible that the high familial aggregation estimates stem from environmental influences such as early music training, which has been identified as a key environmental determinant of AP (Sergeant, 1969; Miyazaki, 1988; Profita and Bidder, 1988; Gregersen et al., 2007; Wilson et al., 2012).

In view of this, Baharloo et al. (1998) controlled for early music training by only analyzing families where the participants and one or more of their siblings had received music training before 6 years of age. The λ_s_ was estimated to be approximately 7.5 (Gregersen, 1998). In a subsequent study, Baharloo and colleagues estimated λ_s_ for the most stringent form of AP, termed “AP-1” (Baharloo et al., 2000). The AP-1 phenotype was characterized by a consistently high level of pitch naming ability, falling at least three standard errors above the mean score of a randomized group of AP and non-AP musicians. Also controlling for early music training, the λ_s_ for AP-1 was estimated to fall within 7.8–15.1, with a greater likelihood of the true value being found near the upper end of this range. It is possible, however, that even after controlling for early music training, the estimated λ_s_ may still be influenced by other shared environmental factors experienced by the AP-1 probands and their concordant siblings. The authors therefore noted that the λ_s_ estimate may not entirely reflect genetic factors. Nonetheless, the high estimates of λ_s_ from various familial aggregation studies suggest a major role for genetic influences on the development of AP and the possibility of a major-gene effect.

More recently, evidence suggests that multiple genetic factors may be involved in the etiology of AP (Theusch and Gitschier, 2011). A segregation analysis performed on 1463 AP-1 probands yielded a segregation ratio of 0.089, which is considerably lower than the estimate from the small AP sample in Profita and Bidder (1988) and the segregation ratios of 0.25 and 0.5 for autosomal recessive and autosomal dominant inheritance, respectively. This suggests that AP was not inherited in a simple Mendelian fashion, however, genetic factors likely play a role since within this larger sample, 11 out of 14 MZ twin pairs were concordant for AP-1 in comparison to only 14 out of 31 DZ twin pairs. These results yielded a significantly different casewise concordance rate of 78.6 and 45.2%, respectively.

Other supporting evidence for a genetic basis for AP comes from ethnicity effects observed in music students. Gregersen et al. (2001) reported that Chinese, Korean and Japanese music theory students had a substantially higher incidence of AP (47.5%) compared to Caucasian students (9%). Although some researchers have attributed this “Asian advantage” to environmental factors, such as early tone-language exposure (Henthorn and Deutsch, 2007), this does not fully account for the higher incidence of AP among all Asian ethnic subgroups since essentially, Korean and Japanese are not tone languages (Sohn, 1999; Zatorre, 2003; Kubozono, 2012). Further analysis of Gregersen's et al. (2001) study data revealed that the age of onset of music training and exposure to a “fixed do” training method before the age of 7 were the only strong predictors of AP acquisition in this sample (Gregersen et al., 2007).

Possible ethnicity effects for AP can also be observed from a genome-wide linkage study of 73 families of European, East Asian, Ashkenazi Jewish and Indian descent (Theusch et al., 2009) in the United States and Canada. In each family there were at least two AP possessors, not limited to a parent-child relative pair. Non-parametric multipoint linkage analyses were suggestive of linkage on chromosomes 8q24.21 (LOD = 2.330) and 8q21.11 (LOD = 2.069) for the European/Ashkenazi Jewish/Indian combined dataset (Table 3). Notably, one of the four genes found near the linkage peak on 8q24.21 was *ADCY8* (adenylate cyclase 8), which is expressed almost exclusively in the brain and is implicated in learning and memory processes (Wong et al., 1999; Ludwig and Seuwen, 2002; De Quervain and Papassotiropoulos, 2006). When only the subset of 45 AP families of European descent was examined, there was strong evidence of linkage on chromosome 8q24.21 (LOD = 3.464 at SNP rs3057), suggesting that at least one gene within this linkage region could predispose AP in individuals of European descent. A number of other linkage peaks were found in the European families, namely on loci 8q21.11 (LOD = 2.236), 7q22.3 (LOD = 2.074) and 9p21.3 (LOD = 2.048). These peaks suggest that different genetic factors may underpin the etiology of AP, even within the same population. The linkage region on 7q22.3 was also observed in a subset of 19 AP families of East Asian ancestry, albeit with a smaller linkage peak (LOD approximately between 1 and 1.5). These findings support a strong genetic contribution to AP, which is likely to be heterogeneous. In other words, AP may be predisposed by different genetic variants at different chromosomal regions, both within and across populations of different ancestries.

A recently published study investigated the genetic relationship between AP and synaesthesia through a combined genome-wide linkage analysis of 53 multiplex families with AP (i.e., families in which multiple family members have AP) and 36 multiplex families with synaesthesia (Gregersen et al., 2013). Interestingly, 28 of the 126 AP possessors from the AP families reported synaesthesia, while eight synaesthesia families had a member with AP. Separate non-parametric linkage analysis of the AP and synaesthesia datasets revealed overlaps in several linkage regions (LOD > 2), especially on chromosomes 2 and 6. Given this overlap and the hypothesis that the two phenotypes may be jointly influenced by genes underpinning brain structural and functional connectivity, the researchers combined the AP and synaesthesia datasets for analysis. This revealed significant linkage on chromosome 6q14.1–6q16.1 (LOD = 4.68), where notably, a small linkage peak (LOD = 1.72) had been reported for the subset of 45 AP families of European ancestry studied by Theusch et al. (2009). Upon sequencing several potential candidate genes in this region, Gregersen et al. found that AP possessors from four of the AP multiplex families shared one or more of three **non-synonymous variants** of the gene *EPHA7* (Ephrin type-A receptor 7). *EPHA7* has been implicated in brain development, particularly establishing neural connectivity between auditory cortex and other cortical regions with the thalamus (North et al., 2013; Torii et al., 2013). Since neuroimaging studies have reported that both AP and synaesthesia are marked by atypical structural and functional connectivity (Rouw and Scholte, 2007; Loui et al., 2011; Dovern et al., 2012), it is conceivable that *EPHA7* variants may influence these two phenotypes. More research involving extensive resequencing on the *EPHA7* gene is needed in order to confirm its involvement. Using parametric linkage analysis, a more complex pattern of linkage was also observed on chromosome 2 in the combined AP and synaesthesia dataset, with a heterogeneity LOD score of 4.7 at SNP rs1482308. When only the AP families were considered, a maximum heterogeneity LOD score of 3.93 was observed at SNP rs6759330 on chromosome 2.

Inherent neuroanatomical differences between AP and non-AP possessors may also be genetically influenced. AP possessors show increased leftward asymmetry of the planum temporale (PT) due to a significantly smaller right mean PT volume compared with non-AP possessors (Keenan et al., 2001; Wilson et al., 2009). Keenan et al. (2001) suggested that the “pruning” of the right PT may be prenatally determined rather than due to early music training, since non-AP musicians with early music training do not manifest similar asymmetry. Wilson et al. (2009) subsequently demonstrated a striking difference in the right mean PT volumes of musicians with AP and quasi-absolute pitch (participants who scored between 20 and 90% on a note-naming test), even though the age of onset of music training did not differ significantly between these groups. Other supporting evidence comes from the discovery of an adult AP possessor, R.M., who had minimal music training but was able to perform a pitch memory task at a level indistinguishable from AP musicians. This case indicates that an early onset of music training (or any music training) may not be essential for AP to emerge (Ross et al., 2003), suggesting that AP and non-AP possessors may be using different pitch processing mechanisms (McLachlan et al., 2013b) that in part, reflect genetically influenced neuroanatomical differences.

#### Congenital amusia

Congenital amusia (commonly known as “tone deafness”) is a fine-grained pitch perception deficit characterized by the inability to detect “wrong” notes in melodies, despite normal intellect, language and hearing abilities (Peretz and Hyde, 2003). Congenital amusia is uncommon in the general population, with an estimated population prevalence of 4% (Kalmus and Fry, 1980). While the neurological basis of congenital amusia has been well-investigated (Peretz and Hyde, 2003; Hyde et al., 2007; Mandell et al., 2007; Loui et al., 2009; Mignault Goulet et al., 2012), few studies have explored its genetic basis.

In the first familial aggregation study on congenital amusia, Peretz et al. (2007) administered an online amusic diagnostic test to 13 amusic probands and 17 controls, as well as 58 family members of the probands (from 9 large families) and 58 family members of the controls (from 10 families). The results showed that 39% of first-degree relatives have congenital amusia, whereas only 3% of controls were similarly diagnosed. Notably, the λ_s_ for congenital amusia was 10.8, whereas the offspring recurrence risk was much lower at 2.3. While the high λ_s_ suggests a probable genetic basis for congenital amusia, Peretz et al. speculated that exposure to an enriched musical environment may mitigate the risk for offspring of amusic probands. However, Mignault Goulet et al. (2012) reported that after four weeks of daily music listening, the music perception scores and electrophysiological measures of seven amusic children (aged 10–12 years) did not vary essentially. This suggests that daily music listening is insufficient to improve pitch perception performance or stimulate neural plasticity in amusic children.

#### Music perception: pitch, rhythm and sound patterns

While music training may be necessary to develop specific music skills, there exists a “commonplace musical competence” that is possessed or easily acquired by most (Trehub, 2003). Investigations of infant musical behavior have shown that infants are capable of detecting melodic or rhythmic changes in musical patterns, as well as perceiving changes in pitch and rhythm (Trehub et al., 1984, 1987, 1999; Trainor and Trehub, 1992, 1993; Zentner and Kagan, 1996; Trainor and Heinmiller, 1998; Trainor et al., 2002; Trehub, 2006; He and Trainor, 2009) (Honing et al., 2009; Winkler et al., 2009). Coupled with the ubiquity of music across all cultures (McDermott and Hauser, 2005), these findings suggest that all humans are endowed with an intrinsic form of musicality, and that genetic factors may play a role in its manifestation.

In particular, individual differences in the ease of auditory skill acquisition point to predisposed differences in auditory ability. In one study, participants were classified as slow or fast learners in an auditory discrimination training task. Differences in behavioral performance were reflected in differential patterns of training-induced functional activation between the two groups (Gaab et al., 2006). Compared with the slow learners, fast learners recruited the left supramarginal gyrus and left Heschl's gyrus to a greater extent during the post-training phase. Jäncke et al. (2001) obtained similar findings, with different short-term functional activation patterns for participants who improved at a frequency discrimination task compared to those who showed no improvement. Likewise, Zatorre et al. (2012) found that participants who learned a micromelody task more quickly had steeper fMRI BOLD responses to pitch changes in their auditory cortex, even before they trained on the task. These findings suggest that predisposed differences in brain functioning may influence an individual's music perception abilities and the capacity to acquire musical skills.

Relative pitch (RP) perception may also be genetically influenced, as inferred from an ethnicity study by Hove et al. (2010). These researchers examined the RP ability of secondary school students with minimal music background using an interval identification task. Students from China or Taiwan (mean score = 72%) significantly outperformed Caucasian (mean score = 45.5%) and Hmong students (mean score = 45.5%), even though both Mandarin and Hmong are tonal languages. The researchers then conducted a similar study with Caucasian, Chinese, and Korean undergraduates with minimal music training (Hove et al., 2010). The Chinese (mean score = 72.2%) and Korean students (mean score = 78.2%) significantly outperformed the Caucasian students (mean score = 58.2%) but performed similarly to each other. Interestingly, these ethnicity effects only occurred in the pitch domain with no differences observed on a rhythm-pattern task. Moreover, as most of the Korean participants spoke Seoul or standard South Korean, both of which are non-pitch-accented (Sohn, 1999), it is unlikely that the ethnicity effects stemmed from tone-language experience. Neither the degree of tone-language experience (fluent or non-fluent), the primary language spoken at home (tonal or non-tonal), nor time spent in East Asia during early childhood were associated with RP ability of the East Asian participants. It is therefore possible that the ethnicity effects observed for RP processing have a genetic basis.

Foster and Zatorre (2010) observed that gray matter volume and cortical thickness in the right Heschl's sulcus and bilateral intraparietal sulcus predicted performance on a relative pitch task, even after accounting for music training. As significant heritabilities (65–97%) for overall brain volume and gray and white-matter volumes have been consistently documented across behavioral genetics research (Peper et al., 2007), these findings are consistent with genetic influences on RP processing. In addition, a longitudinal twin study reported considerable genetic influences (up to 56% heritability) on structural plasticity in the frontal and temporal cortices (Brans et al., 2010). Although music training-induced structural neuroplasticity has been well-documented (see in this Research topic Barrett et al., 2013 and Merrett et al., 2013), this finding suggests that structural plasticity effects may also be genetically influenced.

In a large twin study conducted in 2001, 136 MZ twin pairs and 148 DZ twin pairs undertook the Distorted Tunes Test (DTT), in which they judged whether simple well-known melodies contained incorrect pitches that rendered them “out-of-tune” (Drayna et al., 2001). Twin structural modeling revealed a very high heritability estimate of 71–80% with no effect of shared environment, thus indicating a substantial genetic component influencing melodic perception ability.

A study conducted on 15 musical Finnish families investigated the genetic basis of music aptitude using three widely-used music perception tests: the Karma Music Test, and Seashore's pitch and rhythm discrimination tests (Pulli et al., 2008). The Seashore tasks use paired discrimination to assess pitch and rhythm perception (Radocy and Boyle, 2012), while the Karma Music Test assesses the ability to recognize patterns in sound sequences (Karma, 2007). Heritability estimates of 42, 57, 21, and 48% were obtained for the Karma Music Test, Seashore's pitch and rhythm discrimination tests, and the combined score on all three tests, respectively. Genome-wide linkage analysis revealed significant evidence of linkage on chromosome 4q22 (LOD = 3.33 near markers D4S423 and D4S2460) and suggestive linkage evidence on chromosome region 8q13-21 (LOD = 2.29) for the combined score. Interestingly, the suggestive linkage peak at 8q13-21 was close to the linkage on chromosome 8q21.11 identified in the AP study by Theusch et al. (2009), pointing to a possible convergence of AP and general music perception abilities. A possible candidate gene at the tallest linkage peak of chromosome 4q22 is the netrin receptor *UNC5C*. Netrins are proteins that direct axon extension and cell migration during neural development, with studies showing interactions between netrins and robo family receptors (Stein and Tessier-Lavigne, 2001). One such receptor, *ROBO1*, is a candidate gene for dyslexia (Carrion-Castillo et al., 2013). One of the genes found on 8q13-21 is *TRPA1*, which was proposed as a non-essential subunit of the hair-cell transduction channel in the vertebrate inner ear (Corey, 2006). The authors of this study posited that the low selection pressure of *TRPA1* may make it susceptible to mutations and perhaps lead to variability in the sound perception ability of individuals. Taken together, these linkage results suggest a genetic contribution to music perception underpinned by several predisposing genes on 4q and 8q (Pulli et al., 2008).

A follow-up candidate gene study involving 19 musical Finnish families found that the *AVPR1A* (arginine vasopressin 1a) **haplotype** RS1+RS3 on chromosome 12q has significant associations with performance on the Karma Music Test and the combined score on the Karma and Seashore music tasks (Ukkola et al., 2009). Analyses on the **polymorphisms** of other candidate genes such as *SLC6A4*, *TPH1*, and *DRD2* yielded weak and inconclusive results. Previous studies have shown that arginine vasopressin (AVP) plays a key role in social cognition and behavior (Ferguson et al., 2002; Bielsky et al., 2004; Depue and Morrone-Strupinsky, 2005; Hammock and Young, 2005) and in social and spatial memory (Aarde and Jentsch, 2006). Its association with auditory pattern perception in this study suggests a potential link between music perception and human social functioning.

Using the same music perception measures as the two aforementioned studies, a recent study analyzed genome-wide CNVs in five multigenerational Finnish families and in 172 unrelated individuals (Ukkola-Vuoti et al., 2013). The CNV analysis detected several copy number variable regions (CNVRs) containing genes that influence neurodevelopment, learning and memory. Notably, a deletion on 5q31.1 was present in some participants who obtained a low combined score on the Karma and Seashore music tasks, accounting for 54% of the low-scoring individuals from two families and 7% of low-scoring unrelated participants. This particular CNVR covers the protocadherin-α gene cluster (*Pcdha* 1-9), which is implicated in neural migration, differentiation, and synaptogenesis, as well as learning and memory (Fukuda et al., 2008). Since learning and memory are crucial to music skill acquisition, including music perception (McLachlan et al., 2013a), the authors proposed that *Pcdha* may be a potential candidate gene influencing music perception and practice. Also noteworthy was the identification of a novel large 1.3**Mb** duplication on 8q24.22 in an individual with a low combined score. This region was previously reported as a major linkage region for AP (Theusch et al., 2009). As large duplications may have detrimental effects on neurodevelopment (Almal and Padh, 2012; Grayton et al., 2012), the authors speculated that a duplication in this region may have negatively impacted the participant's pitch perception accuracy. Due to the relatively small sample size and a lack of screening for neurocognitive deficits, the authors acknowledge that these results are preliminary, and there remains a possibility that the identified CNVs may not be predisposing for music perception *per se*.

In the first study to demonstrate a link between music perception and genes that are expressed in the auditory pathway, the same Finnish research group conducted a large-scale genome-wide linkage and association study on the music perception abilities of 767 people from 76 Finnish families (Oikkonen et al., 2014). Participants were again assessed using the Karma Music Test and the Seashore pitch and rhythm discrimination tasks, with estimated heritabilities for each test and the combined test score reported as 46, 68, 21, and 60%, respectively. While the heritability estimates for the Karma Music Test and the rhythm discrimination task are similar to the estimates reported by Pulli et al. (2008), the estimate for pitch discrimination is higher, which in turn may have inflated the heritability estimate for the combined test score. SNP linkage and association analyses uncovered multiple chromosomal regions containing auditory pathway genes. Specifically, the strongest association was observed on 3q21.3 (at SNP rs9854612) for the combined test score. Located close to 3q21.3 is the *GATA2* (GATA binding protein 2) gene, which has been implicated in the development of the inner ear (Haugas et al., 2010) and the inferior colliculus (Lahti et al., 2013). The inferior colliculus is a key structure in the peripheral auditory pathways that supports the initial integration of pitch, direction and loudness information necessary for music perception (McLachlan and Wilson, 2010). Linkage analysis also revealed several linkage regions on chromosome 4, spanning 4p15 to 4q24. The strongest linkage was observed for the pitch discrimination task on chromosome 4p14, which is located next to the *PCDH7* (protocadherin 7) gene. Notably, two SNPs (rs13146789 and rs13109270) of *PCDH7* also showed strong associations with the combined test score. *PCDH7* is expressed in the developing cochlea of the chicken and the amygdala of the mouse (Hertel et al., 2012; Lin et al., 2012), providing possible support for its role in music perception. Finally, some evidence of linkage was found on 4q21.23-22.1 and 4q24 for performance of the Karma Music Test, replicating the significant linkage on 4q22 reported by Pulli et al. (2008) for the combined test score. The current study, however, did not replicate the previously reported association between *AVPR1A* and music perception (Ukkola et al., 2009), nor did it observe any linkage or association evidence on 8q24.21, the putative AP region (Theusch et al., 2009).

In the rhythm domain, one study has reported that mutation of the *FOXP2* (Forkhead box protein P2) gene on chromosome 7q31 impairs rhythm perception and production, while leaving pitch perception and production abilities intact (Alcock et al., 2000). As *FOXP2* has been implicated in an inherited speech and language disorder (Lai et al., 2001), these findings suggest a possible shared genetic basis for speech and rhythm, while pitch-based music abilities are likely influenced by other genetic factors (Peretz, 2009).

#### Music memory

There is evidence that six to eight-month old infants have already developed long-term memories for music and are able to distinguish between familiar and novel music (Saffran et al., 2000; Plantinga and Trainor, 2003). In addition, exposure to melodies presented prenatally for three weeks elicits significant heart rate change in one-month old infants compared to unexposed controls, suggesting that newborn infants are capable of retaining music representations up to six weeks following prenatal exposure (Granier-Deferre et al., 2011). Genetic determinants of memory have been reported in the broader literature, with some studies indicating that memory ability can be predicted by a particular SNP Val66Met variant of the *BDNF* (brain-derived neurotrophic factor) gene (Egan et al., 2003; Hariri et al., 2003). BDNF is evident in the hippocampus (a structure fundamental to new learning and memory) and has been implicated in neuronal growth, survival and maturation, including arborization and synaptic plasticity in the adult brain (Park and Poo, 2012).

In the only genetic study of music memory to date, Granot et al. (2007) investigated the possible association of phonological and music memory with the genes *AVPR1A* and *SLC6A4* (solute carrier family 6 [neurotransmitter transporter serotonin], member 4). The rationale for targeting these two genes included a previously reported relationship between arginine vasopressin (AVP) and spatial and social memory (Ferguson et al., 2002; Aarde and Jentsch, 2006). There is also evidence that serotonin interacts with AVP in the hypothalamus (Albers et al., 2002) and that serotonin increases the secretion of AVP (Gálfi et al., 2005). This points to a possible **epistatic** relationship between the gene *AVPR1A*, which contains the blueprint to synthesize the AVP receptor, and the gene *SLC6A4*, which is the serotonin transporter protein crucial for regulating serotonin supply to serotonin receptors. In view of this, Granot et al. genotyped 82 university students with minimal music training for the *AVPR1A* (RS1 and RS3 haplotypes) and the *SLC6A4* (HTTLPR) polymorphisms using population-based and family-based association analyses. The phonological and music memory performance of the participants were assessed using an extensive battery of tests. Results revealed significant gene by gene epistatic interactions between the *AVPR1A* and *SLC6A4* polymorphisms for two melodic memory tasks, one rhythmic memory task, and one phonological memory task, even after applying conservative Bonferroni corrections for multiple testing. This provides initial evidence for an epistatic relationship between *AVPR1A* and *SLC6A4* polymorphisms that may be linked to short-term memory for music, or more generally, to phonological memory.

#### Music listening

1 In another association study involving *AVPR1A* and *SLC6A4* polymorphisms, Ukkola-Vuoti et al. (2011) investigated the music listening habits of 31 Finnish families using surveys. Family-based association analysis revealed positive associations between *AVPR1A* haplotypes and active music listening. The most significant associations occurred between the RS1+AVR haplotype and current active music listening, as well as the RS1+RS3 haplotype and lifelong active music listening. No significant association was observed between music listening and the *SLC6A4* polymorphisms. In this study, active listening referred to attentive music listening, such as going to concerts. Since the same *AVPR1A* promoter region (RS1+RS3) was shown by Ukkola et al. (2009) to be associated with music perception, these findings suggest a common genetic background for the frequency of active music listening and music perception ability. Moreover, the authors reported that when music perception test scores and music education were covaried with music listening in the association analysis, the significant effect remained, indicating that music listening is independently associated with *AVPR1A*. More broadly, this association suggests that music listening may share common neurobiological pathways with social attachment and communication, given the well-established findings of AVP's mediating role in social behavior (Ferguson et al., 2002; Bielsky et al., 2004; Depue and Morrone-Strupinsky, 2005; Hammock and Young, 2005).

### Music production abilities

#### Singing

Across all cultures humans have a propensity to sing. From two months onwards, infants begin to produce “musical babbling” containing definite music features such as pitch and rhythmic patterns (Welch, 2006). Most children begin imitating songs at approximately age two, by age four they can sing complete songs, and by age 5 most of them can accurately reproduce entire songs (McPherson and Williamon, 2006; Parncutt, 2006).

Although it is likely that variability in children's singing competency is, in part, attributable to environmental factors, such as early music exposure and training, a behavioral study has suggested there may also be an inborn aspect to singing accuracy. Watts et al. (2003) identified individuals who received no professional vocal training yet were described by professional voice teachers as “exhibiting expressed singing talent.” These individuals showed consistently superior performance on pitch-matching tasks, especially in the absence of external auditory feedback, even when compared with trained singers who had at least three years of professional vocal training.

Park et al. (2012) investigated the genetic factors underpinning singing ability by conducting family-based linkage and association analyses on 1008 participants from 73 extended Mongolian families. They administered a pitch production accuracy test and found that 357 of the participants (35.4%) were accurate pitch-matchers, reliably singing the target pitches with deviations less than a semitone. Using pedigree data, the heritability of singing accuracy was reported as 40%. A genome-wide linkage analysis was then conducted, with the most significant linkage peak observed on 4q23 (LOD = 3.1 at marker D4S2986). The findings overlap with regions on chromosome 4q, where there is linkage evidence for music perception ability (Pulli et al., 2008; Oikkonen et al., 2014). A family-based association analysis performed at the putative linkage region revealed that SNP rs12510781 on 4q26 was most significantly associated with singing accuracy. This is an intergenic SNP near the gene *UGT8* (UDP glycosyltransferase 8), whose encoded protein is highly expressed in the brain, especially the substantia nigra (see online Supplementary Figure S2 of Park et al., 2012). The authors also utilized exome sequencing to find other potential candidate SNPs and discovered a non-synonymous SNP (rs4148254) in *UGT8* on 4q26 that was significantly associated with singing accuracy. In addition, CNV analysis using an array comparative genomic hybridization (aCGH) platform showed that a copy number loss at 5.6 kb (5600 base pairs) upstream of *UGT8* may be negatively associated with singing accuracy. Although environmental factors such as education and music training were not considered in this study, the authors argued that because the participants resided in an isolated region with homogeneous culture and most were educated in the same school without additional music training, environmental factors were unlikely to impact greatly on the results. In other words, this study yields evidence that singing accuracy may be heritable in this population and possibly associated with a region on chromosome 4q.

#### Participation in singing activities

Coon and Carey (1989) analyzed the music ability of 11th grade twins by extracting music-related questionnaire data from an earlier study (Loehlin and Nichols, 1976). For participation in singing activities, the heritability estimates were reported as 71% for males and 20% for females, while the corresponding shared environment estimates were 8 and 59% respectively. The significant gender difference in the estimates indicates that a shared environment exerted a stronger effect on females than males, while heritability was much higher in males than females. The authors suggested this may be due to a stereotypical perception that singing is a feminine activity and therefore, males might require greater interest and intrinsic ability to take part in such activities. As this study relied on self-reported data and did not objectively assess the singing ability of the twin pairs, more investigation is warranted to ascertain the genetic contribution to singing ability.

Similar to the association study on music memory by Granot et al. (2007), a candidate gene association study by Morley et al. (2012) tested the relationship between choir membership and allelic variants of the genes *AVPR1A* and *SLC6A4*. An overall association with choral singer status was observed at the STin2 (intron 2) polymorphism in the *SLC6A4* gene, with the STin2 9-repeat and 12-repeat alleles being more common in choral singers, and the 10-repeat alleles more common in non-musically active controls. No significant differences in allele frequencies were observed between the two groups for other *SLC6A4* and *AVPR1A* polymorphisms. Previous studies have reported possible involvement of STin2 in personality traits and reward behavior (Kazantseva et al., 2008; Zhong et al., 2009; Saiz et al., 2010). *SLC6A4* polymorphisms (together with *AVPR1A*) have also been linked to participation in creative dance (Bachner-Melman et al., 2005). As several studies have observed associations between *AVPR1A* polymorphisms and certain music traits (Granot et al., 2007; Ukkola et al., 2009; Ukkola-Vuoti et al., 2011), the non-significant *AVPR1A* association in this study led the authors to speculate that the observed STin2 effect may predispose social behavioral characteristics (i.e., a “predisposed to group activity” phenotype) rather than music ability *per se*.

#### Music creativity

The genetic basis of music creativity was investigated in 19 Finnish musical families using a web-based questionnaire. Participants were asked about their music background and participation in creative music activities, such as music composition, improvisation or arrangement (Ukkola et al., 2009). The findings indicated that creative functions in music may have a strong genetic component in this sample population, with a heritability estimate of 84%. A significant positive association between music creativity and high music perception test scores was also observed. However, no significant associations between music creativity and the polymorphisms of candidate genes such as *TPH1*, *COMT*, and *AVPR1A* were found.

Ukkola-Vuoti et al. (2013) performed a subsequent CNV analysis on five multigenerational Finnish families and 172 unrelated individuals using the same music creativity questionnaire. A “creative phenotype” was characterized by engagement in one or more creative music activities (composing, improvising or music arranging). Results showed that a deletion on 5p15.33 was present in 48% of family members and 28% of unrelated participants who exhibited the creative phenotype, while a duplication on 2p22.1 was present in 27% of creative family members. The region 2p22.1 contains the gene *GALM*, which is associated with serotonin transporter binding potential in the human thalamus (Liu et al., 2011). The medial geniculate nucleus of the thalamus forms part of the auditory pathways, and more generally has been implicated in music-related functions such as beat perception (McAuley et al., 2012), sensorimotor synchronization (Krause et al., 2010) and musical imagery (Goycoolea et al., 2007). Other studies have found a link between the serotonin transporter gene (*SLC6A4*) and music-related functions such as choir participation (Morley et al., 2012) and creative dance (Bachner-Melman et al., 2005). On the other hand, deletions in three CNV regions (2p12, 3p14.1, and 3q28) occurred quite commonly in individuals from two or more families without the creative phenotype, with frequencies ranging from 19 to 31%. The authors acknowledged the preliminary nature of their findings, highlighting their use of uncorrected multiple comparisons. Replication of the findings is clearly warranted, including the use of objective measures of music creativity in future research.

#### Self-reported music ability

In addition to examining participation in singing activities in 11th grade twins, Coon and Carey (1989) used the music-related questionnaire data from Loehlin and Nichols (1976) to investigate other aspects of self-reported music ability, including interest in a music career, participation in music activities, out-of-school music performance experience, and receiving music prizes. Their results showed that while there were genetic influences, the effects of a shared environment were almost always larger across all the variables, with the exception of singing participation (described above) and out-of-school music performances in male twins. For participation in out-of-school music performances, the heritability estimates were reported as 38% for males and 10% for females, while the corresponding shared environment estimates were 18 and 63% respectively. The researchers concluded that music ability is generally more influenced by shared environment than by shared genes in this young adult sample.

Interestingly, contrasting results were obtained in a more recent Netherlands twin study involving 1685 twin pairs aged 12–24 years. Using a self-report questionnaire, this study examined the heritability of domain-specific aptitude (defined as ability within the normal range) and exceptional talent. In particular, the participants were asked to rate their level of competence in various domains such as music, arts, language and sports (Vinkhuyzen et al., 2009). For self-reported music aptitude, the heritability estimates for males and females were 66% and 30% and the shared environment estimates were 8 and 54%, respectively. As for self-reported exceptional music talent, the heritability estimate was 92% with no shared environment effect. The authors concluded that genetic influences possibly account for the variation in aptitude and exceptional talent across domains, including intellect, creativity, and sporting ability to a large extent.

Similar to the music creativity research, both of the above twin studies lacked objective measures of music ability, raising concerns about the reliability of the findings.

## Discussion

As reviewed in this paper, a number of studies have begun to yield insights into the genetic basis of music ability. To date, some promising and converging findings have begun to emerge. Several loci on chromosome 8q have been implicated in more than one music trait. For instance, loci 8q21 and 8q24 have been implicated in AP ability and music perception (Pulli et al., 2008; Theusch et al., 2009; Ukkola-Vuoti et al., 2013). Similarly, loci 4p14 and 4q22 on chromosome 4 have been implicated in music perception, particularly pitch discrimination (Pulli et al., 2008; Oikkonen et al., 2014), while the neighboring locus 4q23 has been implicated in pitch accuracy of singing (Park et al., 2012).

A number of genes have featured quite prominently in music genetics research to date. The gene *AVPR1A* on chromosome 12q has been implicated in music listening (Ukkola-Vuoti et al., 2011), music perception (Ukkola et al., 2009), and music memory (Granot et al., 2007). On the other hand, the gene *SLC6A4* has been associated with music memory (Granot et al., 2007) and choir participation (Morley et al., 2012). The role of *AVPR1A* in social cognition and behavior has been well-investigated, as has the possible interaction between *AVPR1A* and *SLC6A4* in communicative behavior. The associations of these two genes with various music functions raises the intriguing possibility of an overlap in the neurobiological basis of music functions and social behavior.

Replication of the results of existing studies is necessary to confirm the findings, especially in those studies with small sample sizes (e.g., Granot et al., 2007; Pulli et al., 2008; Ukkola-Vuoti et al., 2013). For instance, a recent large genome-wide linkage and association study (Oikkonen et al., 2014) failed to observe an association between *AVPR1A* and music perception, which had previously been reported in a candidate gene study by the same research group (Ukkola et al., 2009). In many of the candidate gene association studies, the polymorphisms of genes such as *AVPR1A* and *SLC6A4* were chosen as candidates based on suggestive results from other music studies. However, the multi-faceted nature of music ability may render a candidate gene associated with one musical function a weak candidate for another musical function. This is illustrated by the study of Morley et al. (2012), which found no association between *AVPR1A* polymorphisms and choir participation despite having adequate statistical power. It may thus be more prudent for researchers to select candidate genes based on supporting evidence from linkage analysis or GWAS of related music abilities. It is also important to note that candidate gene studies have a poor record of replication, with negative findings likely under-reported due to publication bias for positive findings (Ott, 2004; Lewis and Knight, 2012).

In replicating the findings of current studies, it will be important for future studies to use alternate populations and larger samples. It is apparent from Tables 2, 3 that a significant number of molecular genetic studies have been conducted on several multigenerational Finnish families. Extending the findings from these families to other ethnic populations would serve to validate the reported associations. More generally, conducting GWAS in populations of different ancestries has been identified as a key area for future medical genetics research, as the different **linkage disequilibrium** (LD) structure of different populations may help to refine a gene locus of interest (Stranger et al., 2011), and in some cases it may increase the statistical power to detect an association (Pulit et al., 2010). Increased research efforts toward replication will also add to the number of independent molecular genetic studies available for meta-analysis, which in turn, will increase the sample size and statistical power of meta-analyses to detect associations with modest effects (Stranger et al., 2011; Rowe and Tenesa, 2012).

As molecular genetics research in music is still in its infancy, many of the molecular studies reviewed in this paper utilized earlier molecular genetic methods such as linkage mapping or the candidate gene approach. Technological advances now make it possible for commercial arrays to include a combination of SNPs and structural genetic variants (such as CNVs) (McCarroll, 2008). Music genetics researchers can consider integrating these approaches, with findings yielded from CNV analysis able to complement those from SNP analysis (Stranger et al., 2007). Other recent methods include exome sequencing, which provides a more cost-effective and efficient alternative to whole genome sequencing, as well as **methylation studies**, which can be used to investigate the potential contribution of **epigenetic** influences and the underlying molecular and biological mechanisms of a trait (Rowe and Tenesa, 2012). Future studies of the genetic basis of music would therefore likely benefit from a shift toward more current molecular genetic methods to investigate complex traits, especially while new approaches for integrating and analyzing diverse data types are being developed (Battle et al., 2010).

Another important, but as yet under-researched avenue of music genetics research involves exploration of the potential contributions of **epistasis**, gene-environment interactions, and epigenetic influences on music ability. These factors may explain why many of the genetic variants and loci implicated in complex traits could only account for a small percentage of the heritability estimated from family studies (Stranger et al., 2011). Music researchers need to move away from the dichotomous view of nature vs. nurture and develop an awareness of the intricate interplay between genes and environment. For instance, there may be possible genetic influences on ostensibly environmental components such as training-induced neural plasticity (Brans et al., 2010; Vinkhuyzen et al., 2010). Conversely, environmental factors may alter gene expression through epigenetic mechanisms (Fagiolini et al., 2009; Sweatt, 2013).

While the results garnered to date are promising, a more comprehensive investigation of music ability is warranted, including more precise characterization of music phenotypes to identify their genetic basis. As evident from Table 1, the majority of studies have used tests of music aptitude to operationalize music ability, producing an undue emphasis on certain perceptual skills, such as pitch and rhythm discrimination, while ignoring others. This means that other equally important perceptual skills, as well as music production abilities, creativity, sensitivity and expressivity have received minimal investigation. The multifaceted nature of music ability calls for identification and then careful delineation of the range of music phenotypes, with greater research efforts directed toward phenotypes that have been scantly researched. Conceivably, proper characterization of music phenotypes will facilitate identification of relevant genes predisposing these phenotypes through rigorous genetic studies (Levitin, 2012). Music deficits, such as tone deafness (Peretz et al., 2007) and beat deafness (Phillips-Silver et al., 2011), also offer fruitful avenues for further investigation as deficits often have more distinct phenotypic outcomes than abilities. Currently, few genetic studies have focused on music deficits. In addition, a dearth of research effort has been directed toward investigating a possible overlap in the genetic bases of language and music abilities. Our current knowledge is limited to the finding that *FOXP2* may play a role in music rhythm processing as well as language and speech (Alcock et al., 2000; Lai et al., 2001). Comparative genetic research between music and language abilities promises to advance our understanding of the shared and non-shared genetic and neurobiological mechanisms underpinning music and language, and may help elucidate important questions about the origins of music and language (Peretz, 2009).

In conclusion, although currently there is only a handful of research studies in this area, music genetics research has yielded promising preliminary results, highlighting the need for increased research effort in this emerging field. Elucidating the genetic basis of music ability may be challenging due to its multifaceted nature, necessitating careful identification, characterization, and genetic investigation of its many different facets. Coupled with the propitious rate at which molecular genetics and statistical designs are advancing, an increasingly clearer picture of the genetic mechanisms underpinning the etiology of music traits will begin to emerge. These mechanisms may then be linked to established neuroscientific findings of the neurobiological basis of specific music functions and behaviors. Ultimately, this will allow us to gain a deeper understanding of the way in which interactions between nature and nurture shape the development of human music ability over the lifespan.

### Conflict of interest statement

The authors declare that the research was conducted in the absence of any commercial or financial relationships that could be construed as a potential conflict of interest.
