# Supplementary material for: The genetic basis of music ability
Source: Front Psychol. 2014 Jun 27;5:658. doi: 10.3389/fpsyg.2014.00658 (PMC4073543; doi:10.3389/fpsyg.2014.00658)
Supplement: Supplementary file 1 [file DataSheet1.DOCX]

***Supplementary Material***

**The genetic basis of music ability**

**Yi Ting Tan^a*^, Gary E. McPherson^a^, Isabelle Peretz^b^, Samuel F. Berkovic^c^, Sarah J. Wilson^cd^**

1. Melbourne Conservatorium of Music, University of Melbourne, Parkville, VIC, Australia
2. International Laboratory for Brain, Music and Sound Research (BRAMS) and Department of Psychology, Université de Montréal, Montreal, QC, Canada
3. Department of Medicine, Epilepsy Research Centre, University of Melbourne, Heidelberg, VIC, Australia
4. Melbourne School of Psychological Sciences, University of Melbourne, Parkville, VIC, Australia

*** Correspondence:** Ms. Yi Ting Tan, Melbourne Conservatorium of Music, University of Melbourne, Gate 12, Building 141, Royal Parade, Parkville, VIC 3010, Australia

[yitt@student.unimelb.edu.au](mailto:yitt@student.unimelb.edu.au)

1. **Glossary of genetic terminology**

**Additive genetic variance:** The proportion of genetic variation that is the summation of the effects of each individual gene influencing the trait of interest.

**Alleles:** Alternative forms of a gene at a specific position on a **chromosome**. A person inherits two alleles for each gene, one from each parent.

**Allelic variant:** A particular alternate form of an **allele**.

**Array comparative genomic hybridisation (aCGH):** It is a technique which compares a sample of test DNA with the reference DNA to detect copy number gains or losses in the test sample.

**Ascertainment bias:** A biased and non-random sampling method which causes different individuals in the population to have unequal likelihood of being selected as cases. This may cause a systematic distortion in the determination of the actual prevalence of a trait.

**Autosomal dominant inheritance:** A pattern of inheritance in which a **phenotype** can be expressed in individuals who have received just one copy of a particular gene on an autosome (non-sex **chromosome**). An example of a trait with autosomal dominant inheritance is brown eyes.

**Autosomal recessive inheritance:** A pattern of inheritance in which a **phenotype** is expressed only in individuals who have received two copies of a particular gene on an autosome (non-sex **chromosome**). An example of a trait with autosomal recessive inheritance is blue eyes.

**Base pair:** A base pair is akin to a “rung of the **DNA** ladder”. The DNA molecule consists of two strands of DNA, each chemical base on one strand of the DNA is attached to its complement on the other DNA strand by hydrogen bonds, forming a base pair. Of the four chemical bases in the DNA, adenine (A) pairs specifically with thymine (T), whereas cytosine (C) only pairs with guanine (G).

**Candidate gene:** A gene which is suspected to contribute to the trait of interest.

**Chromosome:** A structure containing **DNA** that is found in the nucleus of a cell. Humans have 23 pairs of chromosomes. By convention, the shorter section of the chromosome is called p whereas the longer section is called q.

**Concordance:** Presence of the trait of interest in both twins.

**Copy number:** The number of copies of a particular gene in the **genome**.

**Copy number variants (CNV):** Structural variants (>1,000 **base pair**s) whose **copy numbers** deviate from those found in the human reference **genome**. Smaller structural variants (100-1,000 **base pair**s) are known as indels.

**Dizygotic twins:** Non-identical twins.

**Cryptic relatedness:** The phenomenon where apparently unrelated individuals actually have some unsuspected kinship.

**DNA (deoxyribonucleic acid):** The molecule that contains the genetic instructions to construct and maintain an organism.

**Epigenetic:** Refers to heritable changes to the **genome** that do not change the **DNA** sequence.

**Epistasis:** The interaction between genes at two different **loci**.

**Exome:** The portion of the **genome** containing the **exons**, constitutes 1.5% of the whole genome.

**Exons:** Protein-coding regions of the **genome**.

**Familial aggregation:** The greater prevalence of a trait or disorder among biological relatives compared with the general population. Also known as familial clustering.

**Family-based association study:** A common research design for association study in which relatives of cases serve as controls for the study. The participants are genotyped on a **candidate gene** or **genome** region, and statistical tests are then performed to determine whether the cases have a higher frequency of a particular **genotype** compared with controls, in which case the genotype is likely to play a role in predisposing the trait of interest.

**Genetic heterogeneity**: A phenomenon where apparently identical **phenotypes** are caused by different genes at different **loci.**

**Genetic variant:** A structural change in a gene, e.g. a **CNV**, a **SNP** or an **allelic variant**.

**Genome:** The entire genetic make-up of an organism.

**Genotype:** The genetic makeup of an organism, or group of organisms, usually with reference to a trait of interest.

**Haplotype:** A set of **alleles** (or SNPs) found on the same **chromosome** that are close enough to be inherited together as a unit.

**Heritability:** The proportion of genetic contribution to the trait of interest, with values ranging from 0 (no genetic contribution to the trait) to 1 (the trait is entirely influenced by genetic factors).

**Heritability in the narrow sense (*h^2^*):** The proportion of genetic variation that is due to **additive genetic variance**.

**Heterogeneous phenotype:** A trait characterised by a diversity in the expression of the trait.

**Linkage:** The phenomenon where two gene **loci** in close enough proximity on the same **chromosome** are inherited together.

**Linkage disequilibrium:** The phenomenon where a particular **DNA** **marker** co-occurs with a trait in a population more often than would be expected by chance.

**Locus (plural. loci):** The specific location on a **chromosome** containing the gene of interest, akin to a “street address” for the gene.

**LOD (logarithm of odds) score:** The total relative probability, expressed on a logarithmic scale, that there is evidence of **linkage** among the selected **loci**. A LOD score ≥3 is typically considered significant evidence for linkage.

**Marker:** A **DNA** sequence whose location on a **chromosome** is known. Markers can be used to determine the approximate location of nearby gene(s) that may predispose a trait, although a marker in itself may or may not have known functions.

**Mendelian trait:** Traits predisposed by a single gene.

**Methylation studies:** Studies that evaluate whether methyl groups are attached to **DNA** at cytosine bases of the genes. Such analysis can be used to diagnose conditions in which methylation patterns of the genes may influence the expression of a trait.

**Microarray:** A collection of numerous tiny samples of known DNA sequences typically arranged on a glass slide in a precise grid. They are used to analyse a subject's DNA and to study gene expression.

**Mode of inheritance:** The way a genetic trait is passed from one generation to the next.

**Monozygotic twins:** Identical twins.

**Next-generation sequencing (NGS):** A way of sequencing **DNA** which allows for much higher-throughput sequencing, i.e. many pieces of DNA sequences can be processed simultaneously.

**Non-parametric (model-free) linkage analysis:** A method of **linkage** analysis which tests whether relatives who share resemblance on the trait of interest share more **alleles** than would be expected by chance.

**Non-synonymous variant:** A **genetic variant** that changes the encoded protein.

**Nucleotide:** The basic building block of nucleic acids. A nucleotide consists of a sugar molecule (either ribose in **RNA** or deoxyribose in **DNA**) attached to a phosphate group and a nitrogen-containing base. The bases used in DNA are adenine (A), cytosine (C), guanine (G), and thymine (T). In RNA, the base uracil (U) takes the place of thymine.

**Parametric (model-based) linkage analysis:** A method of linkage analysis which requires the mode of inheritance to be specified. **Linkage** is then tested by comparing the probability of obtaining the current test data if the two **loci** are linked, to the probability of obtaining the test data if two loci are not linked.

**Penetrance:** The extent to which a particular gene or set of genes is expressed in the **phenotypes** of the carriers, i.e. individuals with the specific **genotyp**e. “Complete penetrance” means all the population with the genotype expresses the characteristic phenotype, whereas “incomplete penetrance” means only part of the population with the genotype expresses the phenotype.

**Phenotype:** An observable characteristic of an organism.

**Polymorphism:** A variation in **DNA** sequence which is found in more than 1% of the population

**Population-based association study (or case-control association study):** A common research design for association studies which involves unrelated cases and controls. The participants are genotyped on a **candidate gene** or **genome** region, and statistical tests are performed to determine whether the cases have a higher frequency of a particular **genotype** compared with controls, in which case the genotype is likely to play a role in predisposing the trait of interest.

**Population stratification:** Differences in **allele** frequencies between cases and controls arising from ethnicity differences, rather than the association of genes with trait of interest.

**Proband:** The first person within a family who is ascertained to exhibit the trait of interest.

**RNA (ribonucleic acid):** A molecule similar to **DNA** but single-stranded. RNA transmits genetic information from **DNA** to proteins manufactured by the cells.

**Segregation analysis:** A statistical technique to determine the mode of inheritance of a trait.

**Segregation ratio:** The proportion of offspring who inherit the trait of interest from a parent. Also can be the probability that the offspring will inherit the trait of interest from a parent.

**Sequence capture method:** A method of using high-density microarrays to selectively capture any targeted fraction of the human **genome** (for instance, the **exome**).

**Sibling recurrence-risk ratio (λ_s_):** The proportion of the **probands’** siblings exhibiting the trait of interest, relative to the population prevalence. Similar recurrence-risk ratios can be estimated for other relatives (e.g. offspring). For complex traits underpinned by multiple genetic and environmental factors, reported values of λ_s_ may range from 1.3 to 75, while in **Mendelian** **traits**, λ_s_ values are expected to be much greater (an order of magnitude higher or more).

**Sibling relative risk (sib RR):** The proportion of the **probands**’ siblings exhibiting the trait of interest, relative to the proportion of the controls’ siblings with the trait.

**Single nucleotide polymorphism (SNP):** A common type of variation in **DNA** sequences characterised by a difference in only one **nucleotide**.

**SNP array:** A collection of **SNPs** evenly spaced throughout the human **genome** arranged in a grid pattern on a biochip for simultaneous genetic testing.

**Vertical transmission:** Inheritance of a trait from parent to offspring.
